# Multiserotype Protection Elicited by a Combinatorial Prime-Boost Vaccination Strategy against Bluetongue Virus

**DOI:** 10.1371/journal.pone.0034735

**Published:** 2012-04-13

**Authors:** Eva Calvo-Pinilla, Nicolás Navasa, Juan Anguita, Javier Ortego

**Affiliations:** 1 Centro de Investigación en Sanidad Animal, CISA-INIA, Valdeolmos-Madrid, Spain; 2 Department of Veterinary and Animal Sciences, University of Massachusetts, Amherst, Massachusetts, United States of America; Istituto Superiore di Sanità, Italy

## Abstract

Bluetongue virus (BTV) belongs to the genus *Orbivirus* within the family *Reoviridae*. The development of vector-based vaccines expressing conserved protective antigens results in increased immune activation and could reduce the number of multiserotype vaccinations required, therefore providing a cost-effective product. Recent recombinant DNA technology has allowed the development of novel strategies to develop marker and safe vaccines against BTV. We have now engineered naked DNAs and recombinant modified vaccinia virus Ankara (rMVA) expressing VP2, VP7 and NS1 proteins from BTV-4. IFNAR^(−/−)^ mice inoculated with DNA/rMVA-VP2,-VP7-NS1 in an heterologous prime boost vaccination strategy generated significant levels of antibodies specific of VP2, VP7, and NS1, including those with neutralizing activity against BTV-4. In addition, vaccination stimulated specific CD8^+^ T cell responses against these three BTV proteins. Importantly, the vaccine combination expressing NS1, VP2 and VP7 proteins of BTV-4, elicited sterile protection against a lethal dose of homologous BTV-4 infection. Remarkably, the vaccine induced cross-protection against lethal doses of heterologous BTV-8 and BTV-1 suggesting that the DNA/rMVA-VP2,-VP7,-NS1 marker vaccine is a promising multiserotype vaccine against BTV.

## Introduction

Bluetongue (BT) is a non-contagious, insect-transmitted disease of wild and domestic ruminants that is caused by bluetongue virus (BTV). BTV infection of ruminants occurs throughout much of the tropical and recently also the temperate climate regions of the world, coincident with the distribution of specific species of *Culicoides* biting midges that are the biological vectors of the virus [1], [2]. BTV is the prototype member of the genus Orbivirus of the family *Reoviridae* [3]. After the incursion of BTV into European Mediterranean countries in 1998, vaccination was used in an effort to minimize direct economic losses to animal production, reduce virus circulation and allow safe movements of animals from endemic areas [4]. Live attenuated vaccines are available for different serotypes of BTV. These cell culture adapted viruses, which can also replicate in the vaccinated host, generate significant viremia and neutralizing antibodies, with long-lasting and serotype-specific protection in the recovered animal. Despite their relatively crude nature, inactivated BTV-8 and BTV-1 vaccines are now being used in Europe and they have been effective in preventing further outbreaks of both serotypes.

Although this demonstrates the central importance of vaccination in strategies to combat the spread of BTV, both the modified live and inactivated vaccines are serotype specific. This not only requires a different vaccine for each serotype, but it also invalidates established serological assays (e.g. ELISA) for routine surveillance and import/export testing, since they cannot distinguish between infected and vaccinated animals. Moreover, live BTV vaccines can also become virulent [5] and generate viremia levels that are sufficient for the transmission of the virus to feeding midges [6]. Indeed, several outbreaks of bluetongue in Europe since 1998 were caused by live vaccine strains of BTV-2, 6, 9, 11 and 16. Finally, the circulation of the vaccine strains also results in the exchange of genome segments (reassortment) with field strains, leading to the emergence of novel viruses [7].

Recombinant viral vaccines offer good immunogenicity associated with a replicating virus without the possibility of reassortment. Several prototypic vaccines have been developed and tested with promising results. The capsid proteins VP2, VP5 and VP7 of BTV-1 were expressed in vaccination experiments with a recombinant vaccinia virus, eliciting neutralizing antibody responses and protection against challenge infection [8]. Capripox viruses individually expressing VP2, VP7, NS1 and NS3 of BTV-2 completely prevented viremia in goats challenged with the heterologous serotype three weeks after combined application, but their protective efficacy in sheep was much lower [9]. In another study with homologous virus, a canarypox virus vector expressing both outer capsid proteins of BTV-17 fully protected sheep against virulent challenge [10]. A capripox virus expressing VP7 of BTV-1 even afforded partial protection against a highly virulent heterologous BTV-3 challenge in sheep [11]. In addition, we have described a heterologous prime-boost vaccination strategy against BTV using DNA and recombinant MVA expressing VP2, VP5, and VP7 proteins of BTV-4. We showed that vaccination with these DNA/rMVA of mice deficient in type I IFN receptor (IFNAR−/−), an animal model for BTV infection established in our laboratory [12], [13], were protected against lethal challenge with BTV-4. Moreover, we demonstrated that vaccination induced neutralizing antibodies and T cell mediated immunity [14]. In addition, Virus-like particles (VLPs) of BTV have been produced in insect cell culture using the baculovirus-based protein expression system. VLPs elicit strong and long-lasting immune responses stimulating both B- and T-cell responses [15].

The study of the interaction of BTV with the immune system has showed that neutralizing antibodies [16] and cytotoxic T lymphocytes (CTLs) [17], [18] have a role in protective immunity against BTV [17], [18], [19], [20]. However, in some instances inactivated whole virus vaccines protect against virulent challenge without inducing any measurable neutralizing antibody response. On another hand, the degree of immunity after BTV infection does not always correlate with the level of neutralizing antibodies, even against homologous challenge [21], suggesting the involvement of other factors in the outcome of subsequent infections. Little is known about the location of antigenic determinants for T cells. However, important variations in the protein targets of CTL exist between individuals and host species [22]. VP2 and NS1 are major CTL immunogens in sheep, while VP3, VP5, and VP7 seem to be less immunodominant [23], [24]. However, it has been demonstrated that a BTV-VP7 recombinant capripoxvirus induces significant protective immune responses via a cell-mediated mechanism against homologous challenge. In addition, inactivated BTV-1 vaccine was able to prime sheep to elicit increased cytokine response (Interferon (IFN)γ, Interleukin (IL)-2 and IL-12) against heterologous BTV-23 stimulation *in vitro* [25]. These studies showed a potential role of CTLs in conferring partial heterotypic immunity to BTV-infection in sheep [11], [26]. Overall, these studies demonstrate that the development of vectored vaccines expressing conserved protective antigens results in increased immune activation and would reduce the number of multiserotype vaccinations required.

In this study, we describe a heterologous prime boost vaccination strategy against BTV using DNA/rMVA expressing VP2, VP7, and NS1 proteins (DNA/rMVA-VP2,-VP7, -NS1) of BTV-4. We show that vaccination with these DNA/rMVA of mice deficient in type I IFN receptor (IFNAR^(−/−)^), achieves protective homotypic and heterotypic immunity and protection against homologous and heterologous infection with BTV-4, BTV-8 and BTV-1. These results demonstrate that a rational strategy for the design of effective, cross-protective vaccines involves the combination of antigenic determinants of BTV.

## Results

### Evaluation of protein expression in cells tranfected with cDNAs or infected with recombinant MVAs expressing VP2, VP7, and NS1 proteins from BTV-4

**Figure 1:**
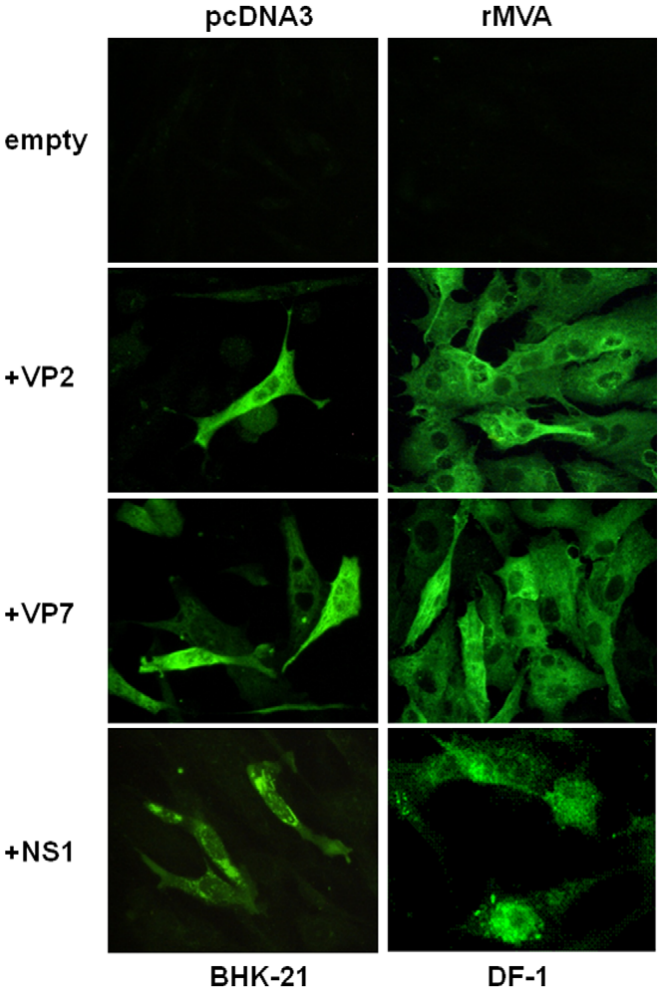
Expression of the VP2, VP7 and NS1 BTV proteins. Immunofluorescence microscopy using a mouse polyclonal antibody against BTV-4 in BHK-21 cells transfected with pcDNA3, pcDNA3-VP2, pcDNA3-VP7, or pcDNA3-NS1 plasmids and DF-1 cells infected with rMVA, rMVA-VP2, rMVA-VP7, or rMVA-NS1.

In order to evaluate the expression of the BTV-4 recombinant VP2, VP7, and NS1 proteins from pcDNA3 and rMVAs vectors in transfected BHK-21 and infected DF-1 cells, respectively, transient expression studies using immunofluorescence microscopy (IFA) were performed. Fluorescence was observed on BHK-21 cells transfected with pcDNA3-VP2, pcDNA3-VP7, and pcDNA3-NS1 but not on cells transfected with the control plasmid pcDNA3 (Fig. 1). Expression of VP2, VP7, and NS1 proteins was also observed in DF-1 cells infected with rMVA-VP2, rMVA-VP7, and rMVA-NS1, respectively, but not in control MVA infected cells (Fig. 1). These data confirm the efficient expression of the proteins from BTV-4 cloned in the DNA and MVA vaccine vectors used for immunization of IFNAR^(−/−)^ mice.

### Heterologous prime boost immunization with pcDNA3-VP2/pcDNA3-VP7/pcDNA3-NS1 and rMVA-VP2/rMVA-VP7/rMVA-NS1 protects IFNAR^(−/−)^ mice against homologous BTV-4 infection

**Figure 2:**
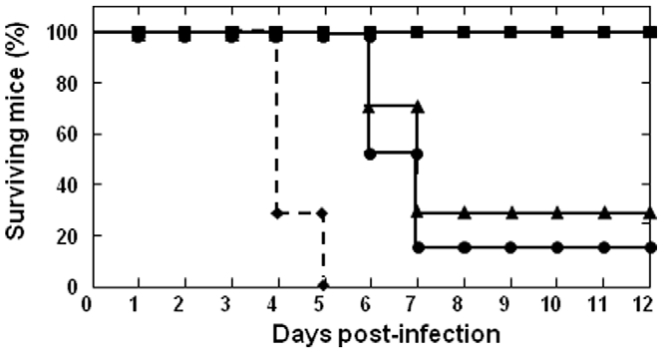
Protection of VP2, VP7 and NS1 vaccinated IFNAR^(−/−)^ mice against a lethal homologous BTV-4 challenge. Mice (8 weeks old, 6 per group) were immunized twice by heterologous prime boost vaccination with pcDNA3 and rMVA expressing VP2/VP7/NS1 (▪), VP2/VP7 (▴), or NS1 (•) BTV-4 proteins (immunized, black line) or pcDNA3 and MVA (♦) (non-immunized, dotted line), administered 2 weeks apart. Two weeks after immunization all mice were intravenously inoculated with 10^3^ PFUs of BTV-4 (lethal dose). Survival rates of immunized and non-immunized IFNAR^(−/−)^ mice after inoculation with BTV-4. The mice were observed every 24 h for 12 days.

Adult IFNAR^(−/−)^ mice were immunized first with pcDNA3-VP2, pcDNA3-VP7 and pcDNA3-NS1 by intramuscular injection. After two weeks, mice were inoculated intraperitoneally with a booster of rMVA-VP2, rMVA-VP7 and rMVA-NS1. Two weeks after the second immunization, immunized and control IFNAR^(−/−)^ mice were challenged subcutaneously with 10^3^ PFUs of BTV-4. While all non-immunized animals died, 100% of the immunized animals did not show clinical signs and were completely protected against lethal challenge (Fig. 2). To analyze whether the combination of the three antigens VP2, VP7 and NS1 is essential for a complete protection against the homologous challenge with BTV-4, two groups of six adult IFNAR^(−/−)^ mice were immunized first with pcDNA3-VP2/VP7 or pcDNA3-NS1 and two weeks later, boosted with rMVA-VP2/VP7 or rMVA-NS1, respectively. In both groups of mice, immunization delayed the appearance of clinical signs and death of the animals upon challenge with BTV-4. In the case of the IFNAR^(−/−)^ mice immunized with vaccine vectors containing VP2 plus VP7, 32% of the animals survived, while immunization with NS1 elicited protection to only 16% of the mice (Fig. 2). These data confirm that the three BTV antigens VP2, VP7, and NS1 are necessary for the total protection against BTV-4 challenge.

The titers of infectious virus recovered in the blood after challenge with BTV-4 were determined in IFNAR^(−/−)^ mice immunized with pcDNA3-VP2/pcDNA3-VP7/pcDNA3-NS1 and rMVA-VP2/rMVA-VP7/rMVA-NS1, and in non-immunized mice by plaque assay. No infectious viruses were detected in the immunized mice; however, titers up to 2,2×10^3^ PFU/ml were observed at 5 days post-challenge in non-immunized animals (data not shown). In addition, the presence of the BTV genome in blood of immunized and non-immunized IFNAR^(−/−)^ mice challenged with BTV-4 was analyzed by RT-qPCR (Table 1). Non-immunized mice (n = 4) were positive for the BTV genome at day three after BTV infection and viremia increased thereafter until the death of the animal. In contrast, all the immunized mice (n = 6) were negative at all the days post-challenge analyzed (*C*t≥38) indicating that protective immunity was achieved after vaccination. These data confirm complete and sterile protection against BTV-4 upon heterologous prime boost immunization by using naked DNA and rMVA as vectors to express VP2, VP7 and NS1 from BTV-4.

**Table 1:** Detection of BTV RNA in blood of immunized and non-immunized IFNAR^(−/−)^ mice after challenge with BTV-4 by RT-qPCR_S5.

Animals	Days post-challenge				
	3	5	7	9	11
**C-1**	33,98	†			
**C-2**	38,79	25.98 †			
**C-3**	23,1	†			
**C-4**	33,65	†			
**I-1**	neg.	neg.	neg.	neg.	neg.
**I-2**	neg.	neg.	neg.	neg.	neg.
**I-3**	neg.	neg.	neg.	neg.	neg.
**I-4**	neg.	neg.	neg.	neg.	neg.
**I-5**	neg.	neg.	neg.	neg.	neg.
**I-6**	neg.	neg.	neg.	neg.	neg.

Results expressed as *C*t and transferred to negative (neg.) according to the cut-off *C*t≥38 described by Toussaint et al. (2007).

I, immunized mice. C, nonimmunized mice.

† , death mice.

### Generation of recombinant VP2, VP7, and NS1 proteins from BTV-4 by using the baculovirus expression system

**Figure 3:**
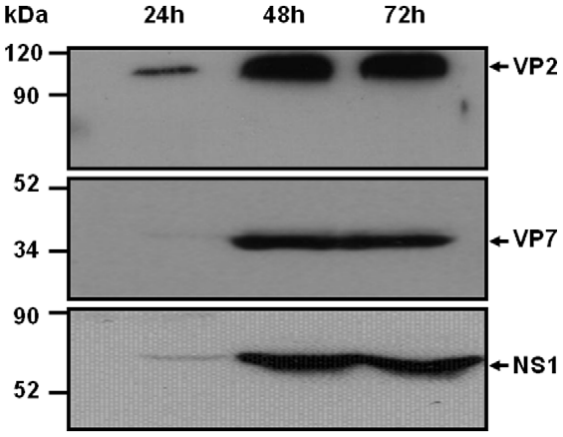
Expression of VP2, VP7, and NS1 proteins in High five cells infected with rBAC-VP2, rBAC-VP7, or rBAC-NS1. High five cells were infected with rBAC-VP2, rBAC-VP7, or rBAC-NS1 and the expression of the proteins was analysed at 24, 48 and 72 hours by western blot using a mouse polyclonal antibody against BTV-4. The position of the three proteins is indicated by an arrow.

To analyse the humoral and cellular immune responses during vaccination, the recombinant VP2, VP7, and NS1 BTV-4 proteins were expressed by using the baculovirus expression system and purified. H5 cells were infected with rBAC-VP2, rBAC-VP7, or rBAC-NS1 and the BTV-4 protein expression was analyzed at 24, 48, and 72 h.p.i by immunoblot. The maximal expression levels of the three proteins were observed at 48 and 72 h.p.i. (Fig. 3). Proteins were purified as described in materials and methods and used to develop ELISA systems that allow the detection of antibodies specific of VP2, VP7, and NS1. In addition, the purified recombinant BTV-4 proteins were used to stimulate splenocytes from immunized mice to analyze the cellular response induced by the vaccines.

### Heterologous prime boost vaccination with pcDNA3/rMVA expressing VP2, VP7, and NS1 induces humoral response against these BTV-4 proteins

**Figure 4:**
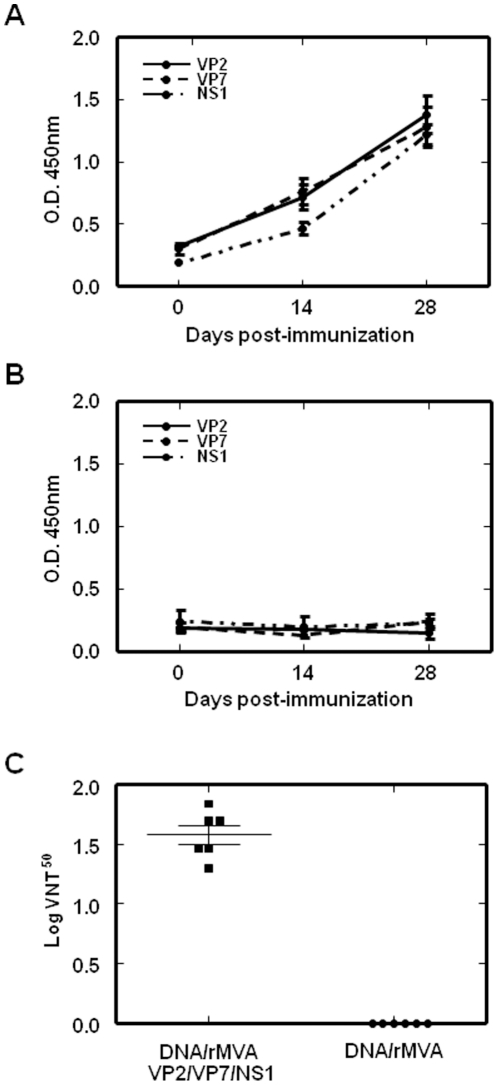
Humoral immune response observed in IFNAR^(−/−)^ mice vaccinated with heterologous prime boost vaccination with pcDNA3 and rMVA expressing VP2/VP7/NS1. The presence of antibodies specific of VP2, VP7, and NS1 in serum of immunized (A) and non-immunized (B) IFNAR^(−/−)^ mice was analyzed by ELISA. Sera from mice immunized with pcDNA3-VP2,-VP7,-NS1 (day 0) and rMVA-VP2,-VP7,-NS1 (day 14) and non-immunized were collected at days 0, 14, and 28 before the challenge with BTV-4, and dilution 1∶50 was analyzed by ELISA as described in Materials and Methods. Standard deviations are shown as error bars. (C) BTV-4 neutralizing antibody detection in VP2/VP7/NS1 immunized mice by VNT. Neutralization titers at day 28 in sera of animals immunized with pcDNA3-VP2,-VP7,-NS1 (day 0) and rMVA-VP2,-VP7,-NS1 (day 14) (▪) and non-immunized (•) are shown. Means are presented (⁃) and standard deviations are shown as error bars.

The presence in serum of specific antibodies to VP2, VP7, and NS1 was analyzed at 14 and 28 days post infection (d.p.i). by ELISA. Antibodies against VP2, VP7, and NS1 were observed at 14 d.p.i, indicating seroconversion. Furthermore, the level of antibodies against these three BTV proteins was highly increased 14 days after the mice were boosted with rMVA-VP2/rMVA-VP7/rMVA-NS1 (Fig. 4A). There were no specific antibody responses in non-immunized mice (Fig. 4B).

In addition, the presence of neutralizing antibodies against BTV in the sera of immunized and non-immunized mice was analyzed by virus neutralization tests. Neutralizing antibodies against BTV-4 were observed in immunized mice 2 weeks after booster treatment with rMVA-VP2, rMVA-VP7, and rMVA-NS1 with a Log VNT^50^ of 1.53±0.15 (Fig. 4C). Neutralizing antibodies against heterologous BTV-1 and BTV-8 were not observed in the serum of immunized mice (VNT^50^≤0.3) at the time analyzed. In addition, neutralizing antibodies against BTV-4, BTV-1, and BTV-8 were not detected in sera from mice immunized with pcDNA3-VP2, pcDNA3-VP7, and pcDNA3-NS1 at 14 days post-immunization and in the non-vaccinated mice.

### Heterologous prime boost vaccination with pcDNA3/rMVA expressing VP2, VP7, and NS1 induces the generation of specific T cell responses

**Figure 5:**
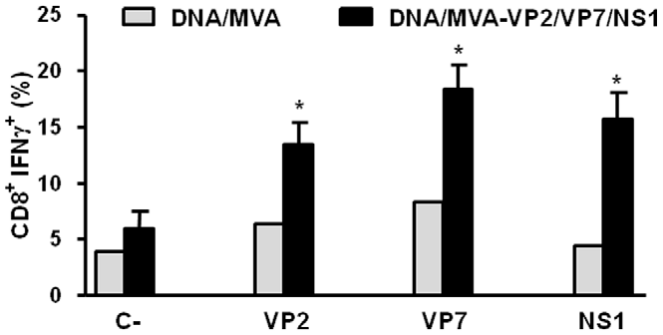
Intracellular staining of IFN-γ, in T CD8+ cells of DNA/rMVA-VP2/VP7/NS1 immunized IFNAR(−/−) mice. Two weeks after second immunization spleens were harvested and the splenocytes were stimulated with 10 µg/ml of recombinant VP2, VP7 or NS1 proteins. At 72 h post-stimulation, intracellular IFN-γ production was analysed in CD8-positive cells by flow cytometry. Results were compared with those of a non-immunized mouse. Grey bars: non-immunized mice; black bars: immunized mice. C-: unrelated stimulus, NS3 BTV protein. The results represent the average of 6 mice ± SD. Asterisks represent significant difference between samples, calculated by Man-Whitney non parametric test (p≤0.01).

We also determined the ability of the triple vaccine formulation to elicit specific T cell responses by intracellular cytokine staining. Whole splenocytes were restimulated with the individual BTV antigens VP2, VP7, and NS1 for 72 h and intracellular IFNγ production by CD8+ T cells was then determined by flow cytometry upon treatment of the cells with the golgi inhibitor, brefeldin A,. VP2, VP7 and NS1 induced the expression of IFNγ by CD8+ T cells upon restimulation (Fig. 5), indicating that all 3 antigens are capable of inducing the activation of CTLs in vivo.

### Cytokine responses in IFNAR^(−/−)^ mice after immunization

**Figure 6:**
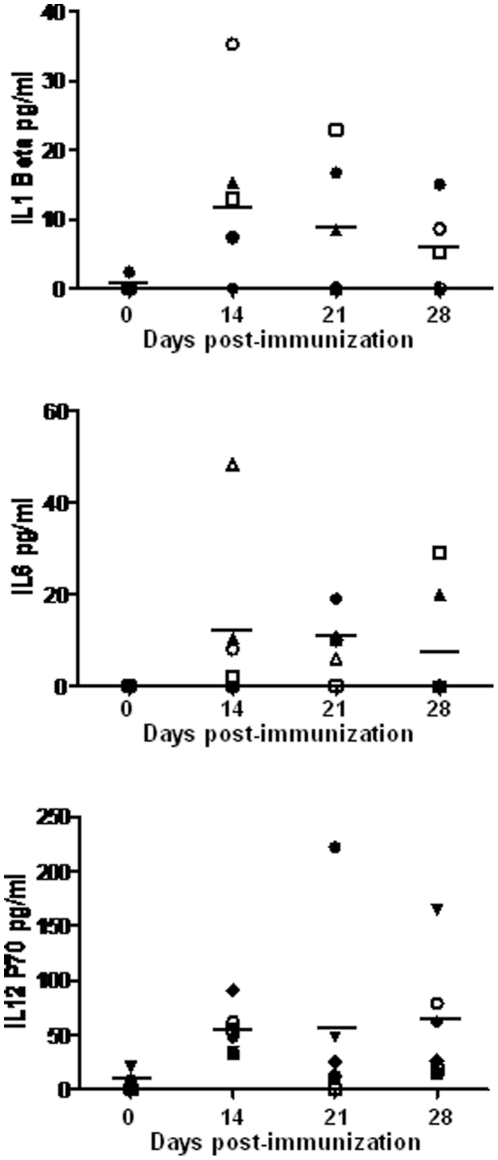
Cytokine responses in IFNAR^(−/−)^ mice after immunization. Sera from mice immunized with pcDNA3-VP2,-VP7,-NS1 (day 0) and rMVA-VP2,-VP7,-NS1 (day 14) (solid symbols) or pcDNA3 (day 0) and MVA (day 14) (symbols) were collected at days 0, 14, 21, and 28. The levels of cytokines were evaluated by a multiplex fluorescent bead immunoassay for quantitative detection of 5 mouse cytokines (Millipore's MILLIPLEX Mouse Cytokine kit). Samples were analyzed with a Luminex 2010 (Luminex Corporation). Means are presented (⁃).

To further analyze the ability of the pcDNA3 and rMVA-based combination vaccine to induce an immune response in the host, three adult IFNAR^(−/−)^ mice were immunized first with pcDNA3 or pcDNA3-VP2, pcDNA3-VP7 and pcDNA3-NS1, and two weeks later with a booster of MVA or rMVA-VP2, rMVA-VP7 and rMVA-NS1, respectively. The presence in serum of the cytokines IL-1β, IL-6, TNF-α, and IL-12 was analyzed at day 14, 21 and 28 post-immunization with DNA, by multiplex cytokine analysis. TNF was not detected in the sera of the immunized mice at the analyzed times. In contrast the presence of IL-1β (13±4.9 pg/ml), IL-6 (11.5±7.5 pg/ml), and IL-12 (54.1±8.7 pg/ml) was observed in the sera at day 14 after immunization with pcDNA containing or not VP2, VP7, and NS1 from BTV-4 (Fig. 6). After the booster with rMVA, the levels in serum of IL-1β and IL-6 were maintained or slightly decreased, whereas the IL-12 levels were slightly increased at days 21 and 28 (68.1±31.1 and 62.6±22.7 pg/ml, respectively) (Fig. 6). All these data support the stimulation of cellular response by the DNA and MVA used as vaccine vectors increasing the efficacy of the vaccines in addition of the BTV antigens.

### Co-transfection of pcDNA3-VP2, pcDNA3-VP7 and pcDNA3-NS1 generates aggregates of the three BTV proteins

**Figure 7:**
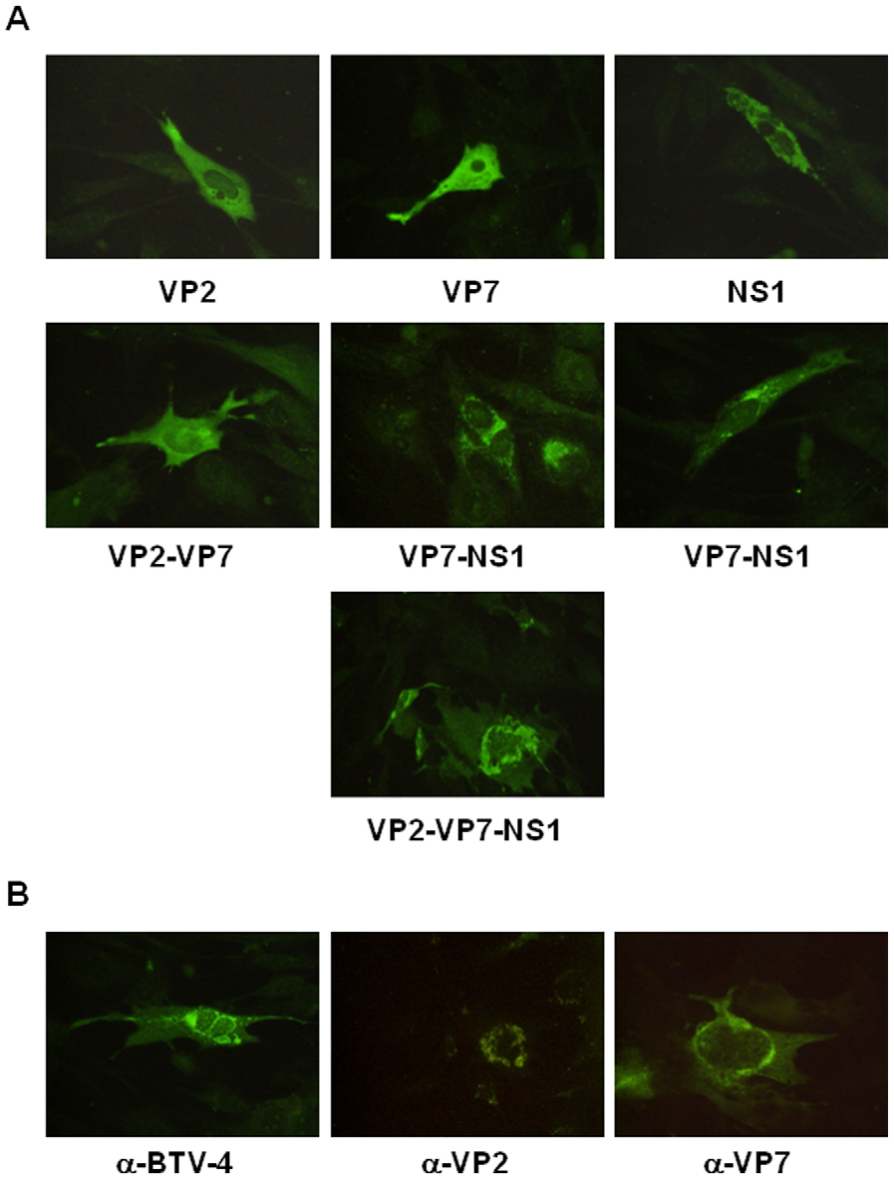
Co-expression of the VP2, VP7 and NS1 BTV proteins. (A) Immunofluorescence microscopy using a mouse polyclonal antibody against BTV-4 in BHK-21 cells transfected with pcDNA3-VP2, pcDNA3-VP7, pcDNA3-NS1 and different combinations of these plasmids. (B) Immunofluorescence microscopy using a mouse polyclonal antibody against BTV-4, or monoclonal antibodies against VP2 and VP7 from BTV-4 in BHK-21 cells transfected with pcDNA3-VP2,-VP7,-NS1.

Particulate immunogens are the best for stimulating both humoral and cellular immune responses [27]. The protein NS1 from BTV forms distinct tubular aggregates in infected or transfected cells. The co-expression of NS1 with VP7 and VP2 could generate aggregates of the three antigens improving the vaccination efficacy. To confirm this possibility BHK-21 cells were transfected with different combinations of pcDNA3-VP2, pcDNA3-VP7, and pcDNA3-NS1 using Lipofectamine 2000 Reagent. After 24 hours, the pattern of expression of the three BTV proteins was analysed by immunofluorescence microscopy using specific BTV-4 antibodies. Fluorescence distributed homegenously in the cytoplasm was observed on BHK-21 cells transfected with pcDNA3-VP2, pcDNA3-VP7, and pcDNA3-VP2+pcDNA3-VP7. In contrast, cells transfected with pcDNA3-NS1 showed a corpusculated signal indicating the aggregation of NS1. Similarly, when NS1 was coexpressed with VP2, VP7 or both antigens, the majority of the signal was also observed in corpusculated forms (Fig. 7A). To confirm that VP2 and VP7 were located in the aggregates generated by NS1, monoclonal antibodies especific for VP2 and VP7 were used in immunofluorencence assays. The results showed that VP2 and VP7 were part of the NS1 aggregates (Fig. 7B) suggesting that the expression of NS1, VP2, and VP7 in the vaccinated animals would generate particulated immunogens that stimulate a better humoral and cellular immune response against BTV.

### Prime boost vaccination with pcDNA3-VP2/pcDNA3-VP7/pcDNA3-NS1 and rMVA-VP2/rMVA-VP7/rMVA-NS1 protects IFNAR^(−/−)^ mice against heterologous BTV-8 and BTV-1 infection

**Figure 8:**
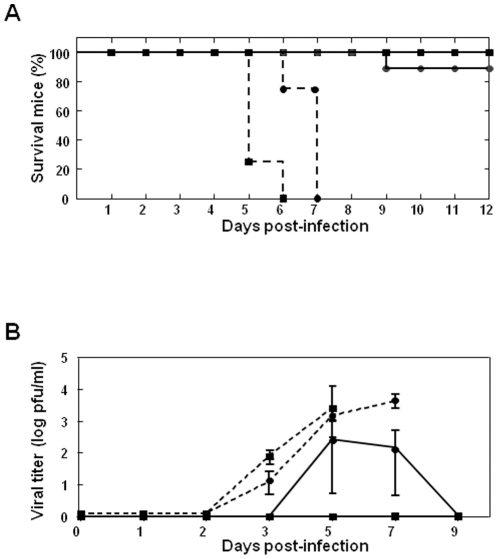
Protection of VP2, VP5 and VP7 vaccinated IFNAR^(−/−)^ mice against lethal BTV-4, BTV-8, and BTV-1 challenges. Mice (8 weeks old, 6 per group) were immunized twice by heterologous prime boost vaccination with DNAs and rMVAs expressing VP2, VP7, and NS1 BTV-4 proteins (immunized, black line) or pcDNA3 and MVA (non-immunized, dotted line), administered 2 weeks apart. Two weeks after immunization mice were intravenously inoculated with 100 PFUs (lethal dose) of BTV-8 (▪) or BTV-1 (•). (A) Survival rates of immunized and non-immunized IFNAR^(−/−)^ mice after inoculation with BTV-8 or BTV-1. The mice were observed every 24 h for 12 days. (B) Titers of BTV-8 (▪) or BTV-1 (•) recovered in blood of immunized and non-immunized IFNAR^(−/−)^ mice after challenge. Virus was extracted from blood and determined as described in Materials and Methods. Each point represents the mean values of the viral titer of six animals, and standard deviations are shown as error bars.

Protein NS1 from BTV shares a high identity in its amino acid sequence among BTV serotypes. The conservation in the sequence of this protein suggests that the strategy of immunization based on VP2, VP7 and NS1 from BTV-4 could also protect against heterologous serotypes. In order to evaluate whether the vaccination strategy based on pcDNA3/rMVA expressing VP2, VP7, and NS1 proteins from BTV-4 also confers protection against heterologous BTV infection, vaccinated IFNAR^(−/−)^ mice were challenged with lethal doses of BTV-8 or BTV-1. Immunized and control IFNAR^(−/−)^ mice were challenged subcutaneously with 10^2^ PFUs of BTV-8 or BTV-1. 100% of the immunized animals were completely protected against the lethal challenge with BTV-8 while 84% survived to the challenge with BTV-1. In contrast, all the non-immunized mice died after challenge with BTV-8 at day 5 and 6 post-infection, or with BTV-1 at day 6 and 7 post-infection, as expected (Fig. 8A). The titers of infectious virus recovered in the blood after challenge with BTV-8 or BTV-1 were determined in immunized and non-immunized IFNAR^(−/−)^ mice by plaque assay (Fig. 8B). No infectious viruses were detected in the immunized mice after challenge with BTV-8; however, a titer of 9×10^2^ PFU/ml was observed in one BTV-1 infected mouse at 7 days post-challenge. The presence of the BTV genome in blood of immunized and non-immunized IFNAR^(−/−)^ mice challenged with BTV-8 and BTV-1 was analyzed by RT-qPCR to detect possible virus replication that can not be determine by plaque assay due to the low sensibility of the assay (Table 2). Non-immunized mice (n = 4) were positive for BTV genome at day three after BTV-8 or BTV-1 infection and the presence of BTV genome increased thereafter until the death of the animal, as was observed in non-immunized mice infected with BTV-4. In contrast, the RT-qPCR reaction yielded no positive results for the majority of the immunized mice (n = 6) challenged with BTV-8 at all days post-challenge analyzed (Ct≥38). Viral genomes were detected in two of the vaccinated mice (Ct = 37) at day five post-challenge. However, in these two immunized mice the Ct value was higher than in the non-immunized mice and the presence of BTV-genomes reverted to negative at day 7 post-challenge. Similar results were observed in the immunized mice (n = 6) challenged with BTV-1, except one mouse that was positive at day 5 post-infection and the presence of BTV genome increased thereafter until the death of the animal. Overall, these data show that vaccination with pcDNA3/rMVA expressing VP2, VP7, and NS1 proteins from BTV-4 exerts protective heterotypic immunity against heterologous infection with BTV-8 or BTV-1.

**Table 2:** Detection of BTV RNA in blood of immunized and non-immunized IFNAR^(−/−)^ mice after challenge with BTV-8 or BTV-1 by RT-qPCR_S5.

Animals	Infection	Days post-challenge				
		3	5	7	9	11
**C-1**	**BTV-8**	31,21	†			
**C-2**	**BTV-8**	35,67	24,89†			
**C-3**	**BTV-8**	32,06	†			
**C-4**	**BTV-8**	37,16	25,06	†		
**I-1**	**BTV-8**	neg.	36,93	neg.	neg.	neg.
**I-2**	**BTV-8**	neg.	neg.	neg.	neg.	neg.
**I-3**	**BTV-8**	neg.	neg.	neg.	neg.	neg.
**I-4**	**BTV-8**	neg.	neg.	neg.	neg.	neg.
**I-5**	**BTV-8**	neg.	37,09	neg.	neg.	neg.
**I-6**	**BTV-8**	neg.	neg.	neg.	neg.	neg.
**C-5**	**BTV-1**	32,05	27,33	23,15†		
**C-6**	**BTV-1**	31,54	26,84	22,98 †		
**C-7**	**BTV-1**	36,15	28,52	†		
**C-8**	**BTV-1**	28,92	25,06	†		
**I-7**	**BTV-1**	neg.	neg.	neg.	neg.	neg.
**I-8**	**BTV-1**	neg.	neg.	neg.	neg.	neg.
**I-9**	**BTV-1**	neg.	37,09	26,81	†	
**I-10**	**BTV-1**	neg.	37,68	neg.	neg.	neg.
**I-11**	**BTV-1**	neg.	neg.	neg.	neg.	neg.
**I-12**	**BTV-1**	neg.	36,63	neg.	neg.	neg.

Results expressed as *C*t and transferred to negative (neg.) according to the cut-off *C*t≥38 described by Toussaint et al. (2007).

I, immunized mice. C, nonimmunized mice.

† , death mice.

## Discussion

The development of recombinant bluetongue vaccines that are inherently safe and compatible with a DIVA (differentiating infected from vaccinated animals) approach has been the subject of research over the last two decades. Vectored vaccines expressing conserved protective antigens can result in increased immune activation and reduce the number of multiserotype vaccinations required, therefore providing a cost-effective product. Recent recombinant DNA technology has provided novel approaches to develop marker and safe vaccines against BTV. We have engineered naked DNAs and recombinant modified vaccinia virus Ankara (rMVA) expressing VP2, VP7 and NS1 proteins from BTV-4. IFNAR^(−/−)^ mice inoculated with DNA/rMVA-VP2,-VP7-NS1 in an heterologous prime boost vaccination strategy generated significant levels of antibodies specific of VP2, VP7, and NS1, and also neutralizing antibodies against BTV-4. In addition, vaccination stimulated specific CD8^+^ T cell responses against these three BTV proteins. Significantly, the vaccine combination expressing NS1, VP2 and VP7 proteins of BTV-4 elicited sterile protection against a lethal dose of homologous BTV-4 infection. Importantly, the vaccine induced cross-protection against lethal doses of heterologous BTV-8 and BTV-1. These results demonstrate that the rational design of vaccine formulations against BTV can promote a multipronged protection that is effective across serotypes.

IFNAR^(−/−)^ mice, a small animal model of BTV, AHSV, and epizootic hemorrhagic disease virus (EHDV) infection [14], [28], [29], has been used in the present work in order to facilitate the studies of protection and immune response conferred by the heterologous prime boost vaccination with DNA and rMVA expressing BTV-4 proteins. Obviously, no experimental mouse model can faithfully capture all aspects of the complex interplay between virus and target host species; however the IFNAR^(−/−)^ adult mouse model has been previously used to investigate the determinants of BTV virulence [30], and to evaluate the protection conferred by BTV and AHSV vaccines [13], [14], [29]. Therefore, our results have important preclinical applications prior to further characterization of the vaccine formulation described herein in the physiological viral host.

Both, neutralizing antibodies [16] and cytotoxic T lymphocytes (CTL) play a role in protective immunity against BTV [19], althought cellular immunity is likely to be crucial to protect against a broad spectrum of BTV serotypes. The rationale for developing DNA vaccines is to deliver the antigen into the MHC class I-processing pathway for the induction of CTLs. On another hand, MVA modulates host immune responses after infection of immature human monocyte-derived dendritic cells [31] by increasing IL-12, IFN-β, TNF-α and IL-6 levels, and Toll-like receptor pathways [32] tending to induce specific CD8+ T cell and strong humoral responses [33]. Our results show that immunizing IFNAR^(−/−)^ mice with empty DNA/rMVA or DNA/rMVA-VP2,-VP7,-NS1 significantly increased their levels of IL-12, IL-1β, and IL-6 in serum. This cytokine response induced by the vaccine vectors fit with the host response induced by BTV infection in ovine conventional dendritic cells where the cytokines IL-12, IL-1β, and IL-6 are up-regulated [34]. The fact that the antiviral host response induced by the vaccine vectors was similar to that induced by infection with BTV supports the suitability of using DNA and rMVA in the development of recombinant BTV vaccines.

Previous work showed that IFNAR^(−/−)^ mice immunized with DNA/rMVA-VP2,-VP5,-VP7 produced good levels of neutralizing antibodies against BTV-4, induced a T cell response, and conferred sterile protection to the IFNAR^(−/−)^ mice against an homologous challenge with BTV-4 [14]. In contrast, this vaccine composition did not conferred good protection against heterologous challenges with BTV-8 (unpublished results). In order to develop a BTV vaccine that generates cross-protection against several BTV serotypes, the protein NS1 was included in the vaccine composition. NS1 is one of the most conserved proteins amongst BTV serotypes [35]. Furthermore, studies with recombinant vaccinia virus expressing BTV proteins to map the location of epitopes recognized by CTLs from Australian merino sheep showed that NS1 was recognized by CTL from all sheep [23]. In addittion, the NS1 gene product on its own assembles into tubules [36] and particulate immunogens are the best for stimulating both humoral and cellular immune responses [27]. Vaccine particulates mimic physiological antigen presentation and create much higher local antigen concentrations than soluble antigen. Additionally, aggregates are protected from degradation, which allows them to remain intact for interaction with antigen-presenting cells and their internalization to appropriate MCH class II-loading compartments, effectively enhancing their presentation of T cells. This therefore results in stronger T cell responses and B cell help, and the overall induction of a stronger immune response [37], [38], [39]. Previous work demostrated that the recombinant tubules were efficiently taken up by antigen-presenting cells and were able to reach the major histocompatibility complex (MHC) class I pathway [40]. The present work shows that the co-expression of VP2, VP7 and NS1 in transfected BHK-21 cells generates aggregates containing the three BTV proteins. This result suggests that in animals immunized with this three proteins particulate immunogens would be generated due to the presence of NS1 that would stimulate a stronger humoral and cellular immune response against BTV, improving the vaccine efficacy.

We now show that IFNAR^(−/−)^ mice immunized with DNA/rMVA-VP2,-VP7,-NS1 produced levels of neutralizing antibodies against BTV-4 similar to those of animals immunized with DNA/rMVA-VP2,-VP5,-VP7 [14], and intracellular cytokine staining showed that VP2, VP7 and NS1 induced the activation of CTLs in vivo. In addition, 100% of the DNA/rMVA-VP2,-VP7,-NS1 vaccinated animals survived to the challenge with BTV-4 and viremia was not observed in the immunized animals after BTV-4 challenge. These results indicate that the heterologous prime boost vaccination with DNA/rMVA-VP2,-VP7,-NS1 confers sterile protection against an homologous challenge with BTV-4 in IFNAR^(−/−)^ mice, as previously observed with DNA/rMVA-VP2,-VP5,-VP7. Interestingly, the vaccination strategy described herein also protected against an heterologus challenge with BTV-1 or BTV-8. Our results show that the inclusion of NS1 in the vaccine composition induces an heterotypic cellular immune response that protects against heterologous BTV infections. There is some evidence of partial cross protection against phylogenetically related serotypes [41]. However, this is the first potential recombinant vaccine against BTV that cross-protects against serotypes that are not related phylogenetically. In addition, this immunization strategy contains DIVA properties. Antibodies against VP2, VP7, and NS1, but not against the other BTV proteins are detected in the sera of DNA/rMVA-VP2,-VP7,-NS1 immunized mice. Recently, a promising marker vaccine was generated by reverse genetics based on BTV-1 with deletions in the essential gene that encodes the VP6 [15]. This vaccine was safe, because it is based on a replication competent and propagation deficient virus, DIVA, and highly protective, but only against homologous challenge. In addition, although VLPs were safe and good DIVA monovalent vaccines against single BTV serotypes when they were used as bivalent immunogens combining BTV-1 and BTV-4, VLPs only protected against BTV-1 probably due to a interference in protective response to BTV-4 [42]. This work raises the question of whether the combinations based on VLPs or inactivated virus could be used as multivalent BTV vaccines. Our results show that the strategy of immunization based on DNA and rMVA expressing VP2, VP7, and NS1 of BTV-4 is the first effective approach to generate a multivalent vaccine against BTV.

In summary, we have developed a marker vaccine and a vaccination strategy against several BTV serotypes based on heterologous prime boost vaccination using DNA and rMVA expressing VP2, VP7, and NS1 proteins of BTV-4. Although further characterization of this vaccine formulation in the virus natural host will be necessary, the data from the IFNAR^(−/−)^ mouse model suggest that the DNA/rMVA-VP2,-VP7,-NS1 marker vaccine could be a promising vaccine against multiple serotypes of BTV.

## Materials and Methods

### Virus and cells

Baby hamster kidney (BHK-21), chicken embryo fibroblast (DF-1), and Vero cells were grown in Dulbecco's modified Eagle's medium (DMEM) supplemented with 2 mM glutamine and 10% fetal calf serum (FCS). Insect cells High Five (Invitrogen) were grown in TC-100 medium supplemented with 10% FCS. BTV serotype 4 (Spain/01) (BTV-4), BTV serotype 8 (Belgium/08) (BTV-8), and BTV serotype 1 (ALG2006/01) (BTV-1) were used in the experiments. Standard virus titrations were performed in Vero cells. Virus stocks were generated by infection of confluent BHK-21 cells using a multiplicity of infection (MOI) of 1. At 48 hours post-infection (h.p.i.), or when total cytopathic effect (CPE) was visible, the cells and supernatants were harvested and centrifuged. The virus were released from the cells by three freeze and thaw cycles. Modified vaccinia virus Ankara (MVA) was growth and titered in DF-1 cells using a MOI of 1.

### Mice

IFN α/βR^o/o^ IFNAR^(−/−)^ mice, on a 129 background were purchased from B&K Universal Ltd. Eight-week old male mice were used throughout. Mice were maintained under pathogen-free conditions and allowed to acclimatize to the biosafety level 3 (BSL3) animal facility at the Centro de Investigación en Sanidad Animal, INIA, Madrid, for 1 week before use in our experiments. All experiments with live animals were performed under the guidelines of the European Community (86/609) and were approved by the ethical review committee at the Centro de Investigación en Sanidad Animal of the Instituto Nacional de Investigaciones Agrarias (CISA-INIA).

### Cloning of NS1 BTV-4 gene and generation of recombinant MVA

Segment 5 corresponding to NS1 was amplified from BTV serotype 4 (Spain/01). To generate pcDNA3-NS1 and the MVA transfer plasmid pSC11-NS1, the restriction site *SmaI* was introduced into the 5′ and 3′ ends of the PCR product. The oligonucleotide primers BTV-4-NS1-SmaI (1) VS (5′-CGC CCG GGA TGG AGC GCT TTT TGA GAA AAT AC-3′), and BTV-4-NS1-SmaI (1659) RS (5′-CGC CCG GGC TAA TAC TCC ATC CAC ATC TG-3′, *Sma I* site underlined), were used to generate a PCR product comprising BTV-4 gene NS1. PCR products were digested with *SmaI* and cloned into the *SmaI* digested pSC11 plasmid or *EcoRV* digested pcDNA3 plasmid to generate pSC11-NS1 and pcDNA-3-NS1, respectively. The generated plasmids were sequenced to analyze the right orientation of the cloned NS1 gene.

The MVA transfer plasmid pSC11-NS1 contained the NS1 BTV gene inserted into the thymidine kinase site of MVA and under the control of the vaccinia virus (VV) early/late promoter p7.5. Recombinant MVA (rMVA) were prepared by infecting DF-1 cells with MVA at a multiplicity of infection of 1 (MOI = 1) and transfecting them with the transfer plasmid pSC11-NS1. Cell cultures were harvested at 48 h.p.i., and the rMVA were selected after plaque assay by the addition of X-Gal to the agar overlay. rMVA was cloned four times by plaque isolation assay and the purity of the rMVA-NS1 analyzed by PCR by using the oligonucleotide primers used to amplify the BTV gene and described above.

The plasmids pcDNA3-VP2 and pcDNA3-VP7, and the rMVA-VP2 and rMVA-VP7 have been previously described [14].

### Generation of recombinant baculovirus (rBAC)

To generate rBAC-NS1, the plasmid pcDNA3-NS1 was digested with *EcoRI* and *Xba I* and the BTV genes were cloned in the *EcoRI* and *Xba I* digested pFastBac-HT-C in order to generate the transfer plasmid pFastBac-HT-C-NS1.

After the generation of the transfer plasmids pFastBac-HT-C-NS1, the recombinant baculovirus rBAC-NS1 was generated by using the Bac-to-Bac Baculovirus Expression System (Invitrogen), according to the method recommended by the manufacturer.

rBAC-NS1 was used to express recombinant NS1 protein from BTV-4 in High five cells. These proteins were purified using the ProBond Purification System (Invitrogen) following the procedure indicated by the manufacturer for protein purification under denaturing conditions. After purification, denatured NS1 was dialyzed overnight in PBS to refold the native structure of the protein.

Recombinant BAC-VP2 and BAC-VP7 were generated as described by Calvo-Pinilla et al. [14].

### Immunoblotting

HighFive cell lysates were analyzed by sodium dodecyl sulfate-polyacrylamide gel electrophoresis (10% polyacrylamide). The proteins were transferred to a nitrocellulose membrane with a Bio-Rad Mini Protean II electroblotting apparatus at 150 mA for 1 h in 25 mM Tris-192 mM glycine buffer (pH 8.3) containing 20% methanol. Membrane binding sites were blocked for 1 h with 5% dried skim milk in TBST (20 mM Tris-HCl [pH 7.5], 150 mM NaCl). The membranes were then incubated with a mouse monoclonal antibody specific for Hys (GE). Bound antibody was detected with horseradish peroxidase-conjugated rabbit anti-mouse antibody and the ECL detection system (Amersham Pharmacia Biotech.).

### Detection of antibodies against VP2, VP7 and NS1 by ELISA

MaxiSorp plates (Nunc, USA) were coated with VP2, VP7, or NS1 purified baculovirus expressed proteins (164 ng per well) and incubated overnight at 4°C. Plates were saturated with blocking buffer (PBS-0.05% Tween 20 and 5% skim milk). The animal sera diluted in blocking buffer were added and incubated for 1 hour at 37°C. After three washes in PBS-0.05% Tween 20, plates were incubated for 1 hour at 37°C with an anti-mouse-HRP secondary antibody (Biorad, USA) at a 1/2,000 dilution in blocking buffer. Finally, after three washes in PBS-0.05% Tween 20, the reaction was developed with 50 µl of substrate solution 3,3′, 5,5′–tetramethylbencidine liquidsupersensitive (TMB) (Sigma) and stopped by adding 50 µl of 3N H_2_SO_4_. Results were expressed as optical densities (ODs) measured at 450 nm.

### Quantification of cytokines in murine sera

IL-12 (p70), IL-6, IL1-β, and TNF were quantified in the sera using the Millipore's MILLIPLEX Mouse Cytokine kit following the procedures indicated by the manufacturer. Samples were analyzed with a Luminex 2010 (Luminex Corporation).

### Immunofluorescence

Cells were plated on glass coverslips and they were infected or transfected. Infections were performed at an MOI of 1 PFU/cell at 37°C in DMEM containing 2% FCS. Free viruses were removed after 90 minutes and the cells were maintained in DMEM 2% FCS. Transfections were performed with lipofectamin 2000 (Invitrogen) with 4 µg of DNA, according to the method recommended by the manufacturer. At the indicated times, the cells were washed with PBS and fixed by addition of 4% paraformaldehyde for 30 min at room temperature. Cells were incubated with a PBS-FCS 20% diluent containing 0.2% Saponin (Superfos-Biosector, Vedback, Denmark) for 1 hour at room temperature. Mouse polyclonal antibody specific for BTV-4 was allowed to adsorb for 90 min at room temperature, and washed three times with PBS-FCS 2%. Cells were then incubated for 30 min at room temperature with an anti-mouse secondary antibody conjugated to Alexa 488. The coverslips were washed five times with PBS-FCS 2%, mounted on glass slides, and analyzed with a Olympus CKX41 microscope.

### Prime boost immunization and challenge with BTV in IFNAR^(−/−)^ mice

Groups of six IFNAR^(−/−)^ mice were immunized by heterologous prime boost vaccination with DNAs and rMVAs expressing BTV-4 proteins or pcDNA3 and MVA (non-immunized mice), administered 2 weeks apart. A suspension of 50 µg of each pcDNA3 construct was administered intramuscularly and 10^7^ PFUs of each rMVA construct were inoculated intraperitoneally. Two weeks after immunization all mice were subcutaneously inoculated with 10^3^ PFUs of BTV-4, or 10^2^ PFUs of BTV-8, or 10^2^ PFUs of BTV-1 (lethal doses) [13]. Mice were bled before each immunization and virus challenged. Sera were tested for BTV-4 neutralizing antibodies by a Virus Neutralization Test (VNT).

### Detection of BTV-4, BTV-8, and BTV-1 in blood

Whole blood was collected in EDTA from all animals at regular intervals after inoculation. The viruses were released from whole blood by three freeze/thaw cycles. The amount of infectious virus was measured by plaque assay on Vero cells or RT-qPCR specific for BTV segment 5.

### BTV-4 neutralizing antibody detection in immunized mice by VNT

The VNT was used to determine neutralizing antibody titers against BTV-4. For plaque reduction assays, 2 fold dilutions of sera were mixed with 100 PFU of BTV-4, incubated for 1 hour at 37°C and then plated onto monolayers of Vero cells. After 1 hour, agar overlays were added and the plates were incubated for 5 days. The titer was determined as the highest dilution that reduced the number of plaques by 50%.

### RT-qPCR specific for BTV segment 5

Whole blood was collected in EDTA from all animals at regular intervals after inoculation and BTV challenge. Total RNA was extracted from blood with TRI Reagent Solution (Ambion), according to the method recommended by the manufacturer. The real-time RT-qPCR specific for BTV segment 5 was performed as described by Toussaint et al. [43].

### FACS analysis

Two weeks after booster immunizations, 10^6^ splenocytes were restimulated with 10 µg/ml of recombinant VP2, VP7 or NS1 proteins or left untreated in the presence of 1 µl/ml of GolgiPlug (BD Biosciences) for the last 6 h of stimulation. At 72 h post-stimulation, the cells were washed with PBS+1%FCS and surface-labelled with a FITC-conjugated anti-CD8 mAb (BD Biosciences) on ice for 30 min. Nonspecific binding of the mAb was blocked by preincubating the cells with Fc Block (2.4G2; BD Biosciences). Intracellular staining for IFNγ (PE-conjugated anti-IFNγ mAb; BD Biosciences) was then performed using the BD Cytofix/Cytoperm Fixation/Permeabilization Kit (BD Biosciences) according to the manufacturer's instructions. Flow cytometry data were collected on an LSR II flow cytometer running the Diva Software (BD Biosciences) and analyzed with FlowJo version 8.6 for MacOS (TriStar).
